# Development of combination adjuvant for efficient T cell and antibody response induction against protein antigen

**DOI:** 10.1371/journal.pone.0254628

**Published:** 2021-08-02

**Authors:** Yasunari Haseda, Lisa Munakata, Chiyo Kimura, Yumi Kinugasa-Katayama, Yasuko Mori, Ryo Suzuki, Taiki Aoshi

**Affiliations:** 1 Vaccine Dynamics Project, BIKEN Innovative Vaccine Research Alliance Laboratories, Research Institute for Microbial Diseases, Osaka University, Suita, Osaka, Japan; 2 Laboratory of Drug and Gene Delivery Research, Faculty of Pharma-Science, Teikyo University, Itabashi-ku, Tokyo, Japan; 3 Department of Cellular Immunology, Research Institute for Microbial Diseases, Osaka University, Suita, Osaka, Japan; 4 Division of Clinical Virology, Center for Infectious Diseases, Kobe University Graduate School of Medicine, Kobe, Japan; National Institute of Animal Biotechnology, INDIA

## Abstract

Most current clinical vaccines work primarily by inducing the production of neutralizing antibodies against pathogens. Vaccine adjuvants that efficiently induce T cell responses to protein antigens need to be developed. In this study, we developed a new combination adjuvant consisting of 1,2-dioleoyl-3-trimethylammonium propane (DOTAP), D35, and an aluminum salt. Among the various combinations tested, the DOTAP/D35/aluminum salt adjuvant induced strong T cell and antibody responses against the model protein antigen with a single immunization. Adjuvant component and model antigen interaction studies in vitro also revealed that the strong mutual interactions among protein antigens and other components were one of the important factors for this efficient immune induction by the novel combination adjuvant. In addition, in vivo imaging of the antigen distribution suggested that the DOTAP component in the combination adjuvant formulation elicited transient antigen accumulation at the draining lymph nodes, possibly by antigen uptake DC migration. These results indicate the potential of the new combination adjuvant as a promising vaccine adjuvant candidate to treat infectious diseases and cancers.

## Introduction

Vaccination is one of the most successful preventive measures against infectious diseases. However, there are still many infectious diseases for which vaccines have not been successfully developed. Most current vaccines work by inducing neutralizing antibodies against pathogens. Clinically efficient T cell immunity-inducing adjuvants for protein antigens are unavailable. Therefore, the development of adjuvants capable of efficient and practical induction of T cell immunity is required [1–3].

Vaccine adjuvants enhance immune responses to antigens [4, 5]. A variety of substances, from small chemicals to oils, can function as adjuvants. These functions can be categorized into two classes: immune-potentiators and delivery systems [6, 7]. Typical immune-potentiators include Toll-like receptor (TLR) agonist ligands such as Poly IC, monophosphoryl lipid A (MPL), and CpG, which stimulate TLR3, TLR4, and TLR9, respectively. TLR ligands originating from pathogens induce innate immune signaling, resulting in the production of interferons and pro-inflammatory cytokine responses, which are essential to elicit adaptive immune responses. A typical delivery system is composed of mineral salts and a variety of oils [8, 9]. Aluminum salt is one of the most commonly used mineral salt adjuvants for clinical vaccines that induce an antibody response [10]. Recently approved new vaccine adjuvants commonly utilize oil emulsions and liposomes, such as AS03, MF59, and AS01 [11, 12]. These delivery system adjuvants deliver antigens to antigen-presenting cells and induce local inflammation at the injection site. However, the exact molecular mechanisms involved have not been completely elucidated [11, 12].

Currently, most clinically available protein vaccines contain a single adjuvant of either an immune-potentiator or a delivery system. However, the combination of multiple adjuvants, especially immune-potentiator plus delivery systems, can synergistically enhance immune responses [13, 14]. AS04 is a clinically approved immune-potentiator plus delivery system combination adjuvant composed of the TLR4 agonist MPL and the aluminum hydroxide mineral salt. Many other combinations that enhance immune responses have also been explored [13, 14].

In this study, we developed a new combination adjuvant consisting of 1,2-dioleoyl-3-trimethylammonium propane (DOTAP), D35, and an aluminum salt. DOTAP is a cationic lipid that is commonly used in nucleic acid transfection reagents [15]. A recent report showed that DOTAP alone is an efficient adjuvant for CD8^+^ T cell response inducing cancer vaccines. However, the mechanisms are not fully understood [16–20]. D35 is a type A CpG oligodeoxynucleotide (ODN) that stimulates TLR9. Four types of CpG ODNs are known: type A, type B, C, and P based on the sequence, backbone chemistry, and resultant differences in biological activity [21, 22]. Type A CpG ODNs (such as D35) have a mostly natural phosphodiester backbone and contain one palindromic sequence. They strongly induce the production of interferon-alpha (IFN-α). In contrast, other type B (such as K3), type-C (such as CpG ODN 2395), and type-P (such as P21889) consist of unnatural phosphorothioate backbones. ODNs with a phosphorothioate backbone all potently induce IL-6. These ODNs also have the potential to induce IFN-α production, which appears to be dependent on the presence of palindromic sequences. Type B ODNs do not contain any palindromic sequence, type-C contains one palindromic sequence, and type-P contains two palindromic sequences. Accordingly, type B is a very poor IFN-α inducer, type-C is intermediate, and type-P is a high IFN-α inducer, with an induction whose level comparable to type A ODNs. Therefore, type A (such as D35) is mostly an IFN-α inducing CpG ODN, type B (such as K3) is mostly an IL-6 inducer, type-C is a high IL-6 and low IFN-α inducer, and type-P potently induces both IFN-α and IL-6 [23]. The adjuvanticity of these four types of CpG ODNs, including D35, has been well demonstrated [24, 25].

Aluminum salts, including Alhydrogel and AdjuPhos, are used in many clinical vaccines as a single adjuvant. Alhydrogel is aluminum hydroxide. It is usually used as an adjuvant for negatively charged protein antigen vaccination because the positive charge of the Alhydrogel in a neutral pH buffer allows electrostatic binding of negatively charged antigens on Alhydrogel, which is necessary for the adjuvant effect [10]. AdjuPhos is aluminum phosphate. It is used as an adjuvant for positively charged protein antigens with similar electrostatic interactions [10].

While DOTAP, CpG, and aluminum salts have demonstrated individual adjuvanticity, the adjuvanticity of these combinations has not yet been fully examined.

In a previous study, we reported that the combination of DOTAP and D35 (DOTAP/D35) is a potent T cell response inducing adjuvant [26]. In this study, we extended this observation and examined various combinations of DOTAP, CpG, and aluminum salts. Our results showed that the addition of aluminum salt to DOTAP/D35 strongly enhanced T cell and antibody responses. We also demonstrated that the physical interaction between antigen protein and other components was an important determinant factor for efficient immune response induction with this combination adjuvant.

## Materials and methods

### Reagents

Low endotoxin ovalbumin (OVA; endotoxin level < 1 EU/mg) (OVA) was purchased from Wako (Osaka, Japan). D35 and K3 were purchased from GeneDesign (Osaka, Japan). P21889 [27] was synthesized by GeneDesign (Osaka, Japan). DOTAP was purchased from Lipoid GmbH (Steinhausen, Switzerland). DOTAP in MES buffer (1 mg/mL) and hen egg lysozyme (HEL) were purchased from Sigma-Aldrich (St. Louis, MO, USA). Alhydrogel (10 mg/mL) and AdjuPhos (5 mg/mL) were purchased from InvivoGen (San Diego, CA, USA). OVA and HEL were dissolved at 10 mg/mL in phosphate-buffered saline (PBS) (14249–24, Nacalai Tesque, Kyoto, Japan). D35, K3, and P21889 were dissolved at 10 mg/mL in water for injection (Otsuka Pharmaceutical, Tokyo, Japan).

### Preparation of DOTAP particles (DOTAP-Nano) by NanoAssemblr

DOTAP-Nano was prepared with a NanoAssemblr Benchtop (Precision NanoSystems Inc., Vancouver, BC, Canada), as previously reported [26]. Briefly, DOTAP was dissolved in ethanol. The lipid solution (10 mg/mL) in ethanol and 25 mM sodium acetate buffer (pH 4.0) was injected into the microfluidic mixer at a volume ratio of 1:3 and a final flow rate of 15 mL/min (3.75 mL/min ethanol, 11.25 mL/min aqueous). The DOTAP-Nano mixtures were immediately dialyzed using dialysis tubing with a molecular weight cutoff (MWCO) of 50 kD (Repligen Corporation, Waltham, MA, USA) against a 5% glucose solution to remove ethanol. DOTAP-Nano was then concentrated to obtain approximately 1.5–2.0 mg/mL DOTAP using Amicon Ultra centrifugal filters (100 kD MWCO, Merck KGaA, Darmstadt, Germany) and sterilized by passage through a 0.22 μm polyvinylidene fluoride filter (Merck KGaA). All lipid nanoparticle preparations were prepared at room temperature. DOTAP-Nano was the default DOTAP in this study. DOTAP in MES buffer used in the experiment described in Figs 4 and 5 was obtained from Sigma-Aldrich.

### Emulsion preparation

Freund’s complete adjuvant (FCA) was purchased from Sigma-Aldrich. To prepare the oil-water emulsion, a GP syringe connector (BrightPath Biotherapeutics, Tokyo, Japan) and NORM-JECT all-plastic lure lock syringes (Henke-Sass Wolf, Tuttlingen, Germany) were used according to the manufacturer’s instructions. Briefly, one syringe was filled with PBS containing the antigen and the other was filled with an equal volume of FCA. The syringes were connected to a GP syringe connector and the plunger was pushed alternately 30 times per minute.

### Mice and preparation of adjuvants

The mice were purchased from CLEA Japan. Female C57BL/6 or BALB/c mice aged 4–10 weeks were used for all experiments. All animal experiments were approved by the Animal Care and Use Committee of the Osaka University and adhered to the regulations of animal experiments at Osaka University (Permit number: Biken-AP-H26-12-2). In most experiments, mice were immunized solely with antigen (OVA or HEL; 10 μg) or antigen plus adjuvants (CpG ODNs, 10 μg; DOTAP, 100 μg; aluminum salt, 40 μg). Typically, DOTAP (100 μg) in 50 μL of 5% glucose solution was mixed with 50 μL of PBS containing OVA (10 μg), D35 (10 μg), and aluminum salt (40 μg). In some cases, 20 mM histidine buffer (pH 6.5) or 20 mM histidine buffer + 0.15 M NaCl were used instead of PBS (Glu/His and Glu/His+NaCl buffer, respectively). Histidine buffers were prepared with 10% sucrose for osmotic pressure control. A total of 100 μL of each mixture was injected intradermally (i.d.) at the tail base (50 μL on the right side and 50 μL on the left side). While using DOTAP in MES (1 mg/mL), minimal amounts of other components were directly added to 100 μL of DOTAP in MES buffer (1 mg/mL) and mixed, for instance, 1 μL of D35 (10 μg), 1 μL of OVA (10 μg), and 4 μL of Alhydrogel (40 μg) or 8 μL of AdjuPhos (40 μg).

### Immunization

In most of the experiments, mice (n = 3 per group) were immunized by i.d. injection of various combinations of adjuvant components and buffers (for details, see each figure legend), at the tail base. The total volume of 100 μL was injected at the right side (50 μL) and left side (50 μL) of the tail base. Seven days after of the sole immunization, the mice were euthanized by cervical dislocation. Serum was collected and spleen cells prepared as described below. OVA-specific antibody titer and OVA-derived peptides (OVA257-264: SIINFEKL for CD8 T cells, OVA323-339: ISQAVHAAHAEINEAGR for CD4 T cells)-specific T cell responses were evaluated by ELISA.

### Splenocyte preparation and in vitro stimulation for ELISA

The mice were euthanized 7 days after one i.d. immunization at the tail base and the spleen was collected from each mouse. A single-cell suspension was prepared. Red blood cells were lysed with ACK lysis buffer (150 mM NH_4_Cl, 10 mM KHCO_3_, 0.1 Na_2_EDTA. The cells were washed with RPMI 1640 and resuspended in R-10 medium (RPMI 1640 medium supplemented with 10% fetal calf serum, 100 units/mL penicillin, and 100 μg/mL streptomycin). Splenocytes were plated in 96-well plates at a density of 2 × 10^6^ cells/well/200 μL. OVA257-264 peptide, OVA323-339 peptide, OVA full protein, HEL107-116 peptide, and HEL full protein 5 μg/mL (final) were added to the cell cultures overnight, incubated at 37°C in 5% CO_2_ incubator. Following centrifugation, the supernatant was collected and examined using mouse interferon-gamma ELISA (Mouse IFN-γ DuoSet ELISA kits; R&D Systems, Minneapolis, MN, USA).

### Serum and antibody titer

Mice were anesthetized using isoflurane 7 days following one i.d. immunization at the tail base. Blood was collected using heparin-coated microcapillaries and centrifuged (6,500 × g, 20 min). The levels of OVA-specific antibody in the plasma were determined using ELISA. All 96-well plates were coated with 100 μg/mL OVA or standard antibodies (total IgG: MBL, Woburn, MA, USA; IgG1 and IgG2c: Southern Biotech, Birmingham, AL, USA) in carbonate buffer (pH 9.6). The wells were blocked with Blockace (KAC Co., Ltd., Kyoto, Japan). Diluted plasma obtained from immunized mice was incubated on the antigen-coated plates. After washing, either goat anti-mouse total IgG, IgG1, or IgG2c conjugated horseradish peroxidase (HRP; Southern Biotech) was added. The mixture was incubated for 2 h at room temperature. After additional washing, the plates were incubated with 3,3′,5,5′-tetramethylbenzidine (TMB; KPL) for 5–15 min. The reaction was stopped with 2N H_2_SO_4_ and the absorbance was measured at 450 nm.

### Interaction between protein and aluminum salt, or D35 and aluminum salt

Soluble protein (10 μg) or protein (10 μg) plus aluminum salt (40 μg) mixtures were centrifuged at 5000 rpm for 3 min. The non-binding protein concentration in the supernatant was measured using a Qubit Protein Assay Kit (Thermo Fisher Scientific, Waltham, MA, USA). Similarly, the D35 (10 μg) plus aluminum salt (40 μg) mixture was centrifuged at 5000 rpm for 3 min. The non-binding nucleic acid concentration in the supernatant was measured using a NanoDrop 2000c device (Thermo Fisher Scientific).

### Interaction between DOTAP and aluminum salt

DOTAP (100 μg) or DOTAP (100 μg) plus aluminum salt (40 μg) mixtures were centrifuged at 5000 rpm for 3 min. The concentration of non-binding DOTAP in the supernatant was measured using a NanoDrop 2000c device (Thermo Fisher Scientific) at an absorbance wavelength of 230 nm. The DOTAP containing buffer solution showed a high absorbance at 230 nm as measured using the NanoDrop 2000c device. However, buffer that did not contain DOTAP demonstrated only a background absorbance at 230 nm (S3 Fig). Thus, the DOTAP concentration could be determined by measuring the absorbance at 230 nm in this assay.

### Interaction assay between D35 and DOTAP, or protein and DOTAP

D35 (10 μg) only, and D35 plus DOTAP (100 μg) mixtures were loaded into Amicon Ultra centrifugal filter units (100 k; Merck Millipore, Burlington, MA, USA) and centrifuged at 14,000 *× g* for 1 min. The D35 concentration in the flow-through (FT) was measured using the NanoDrop 2000c device (Thermo Fisher Scientific). In this assay, DOTAP-bound D35 was trapped using the 100 k filter. Therefore, the FT contained only free D35. Similarly, protein (10 μg) or protein plus DOTAP (100 μg) was loaded into the 100 k Amicon Ultra centrifugal filter units (100 k) (Merck Millipore) and centrifuged at 14,000 *× g* for 1 min. The protein concentration in the FT was measured using a Qubit Protein Assay Kit (Thermo Fisher Scientific).

### Immunization with four different buffers

Mice were immunized with antigen (OVA 10 μg) only or with antigen plus adjuvants (D35, 10 μg; DOTAP, 100 μg; aluminum salt, 40 μg) mixed in four different buffers (Glu/PBS, Glu/His, Glu/His+NaCl, and MES). A total of 100 μL of the particular mixture was injected i.d. at the tail base (50 μL on the right side and 50 μL on the left side).

### Biodistribution assay of OVA antigen

Alexa647-labeled OVA (Thermo Fisher Scientific) was formulated using different combinations of adjuvant components in Glu/PBS buffer. Mice were immunized i.d. at the tail base. After the indicated times, the fluorescent intensities of Alexa647 at the injection site and the draining inguinal lymph node were measured using a VISQUE InVivo Smart-LF in vivo imager (Vieworks, Seoul, South Korea). Mice were administered a low fluorescent diet (iVid-neo; Oriental Yeast, Tokyo, Japan) prior to at least 7 days before the experiment to reduce abdominal background fluorescence as much as possible. This was done because many plant-based ingredients used in regular mouse chows contain chlorophyll components (mainly alfalfa) that emit between 675 and 685 nm, which usually disturbs the signal detection in the abdominal area [28].

### Statistical analysis

Immunization data are presented as the mean ± SD. Statistical significance was calculated using GraphPad Prism 6 software (GraphPad, La Jolla, CA, USA) by a non-parametric method (one-way analysis of variance (ANOVA) with Dunn’s multiple comparison). Adjuvant component binding data are presented as mean ± SD. Statistical significance was calculated using GraphPad Prism 6 software with a parametric unpaired t-test. Significant differences are indicated as ****p<0.0001, ***p<0.001, **p<0.01, and *p<0.05.

## Results

### DOTAP/D35/Alhydrogel combination induces strong T cell response against OVA antigen

**Fig 1:**
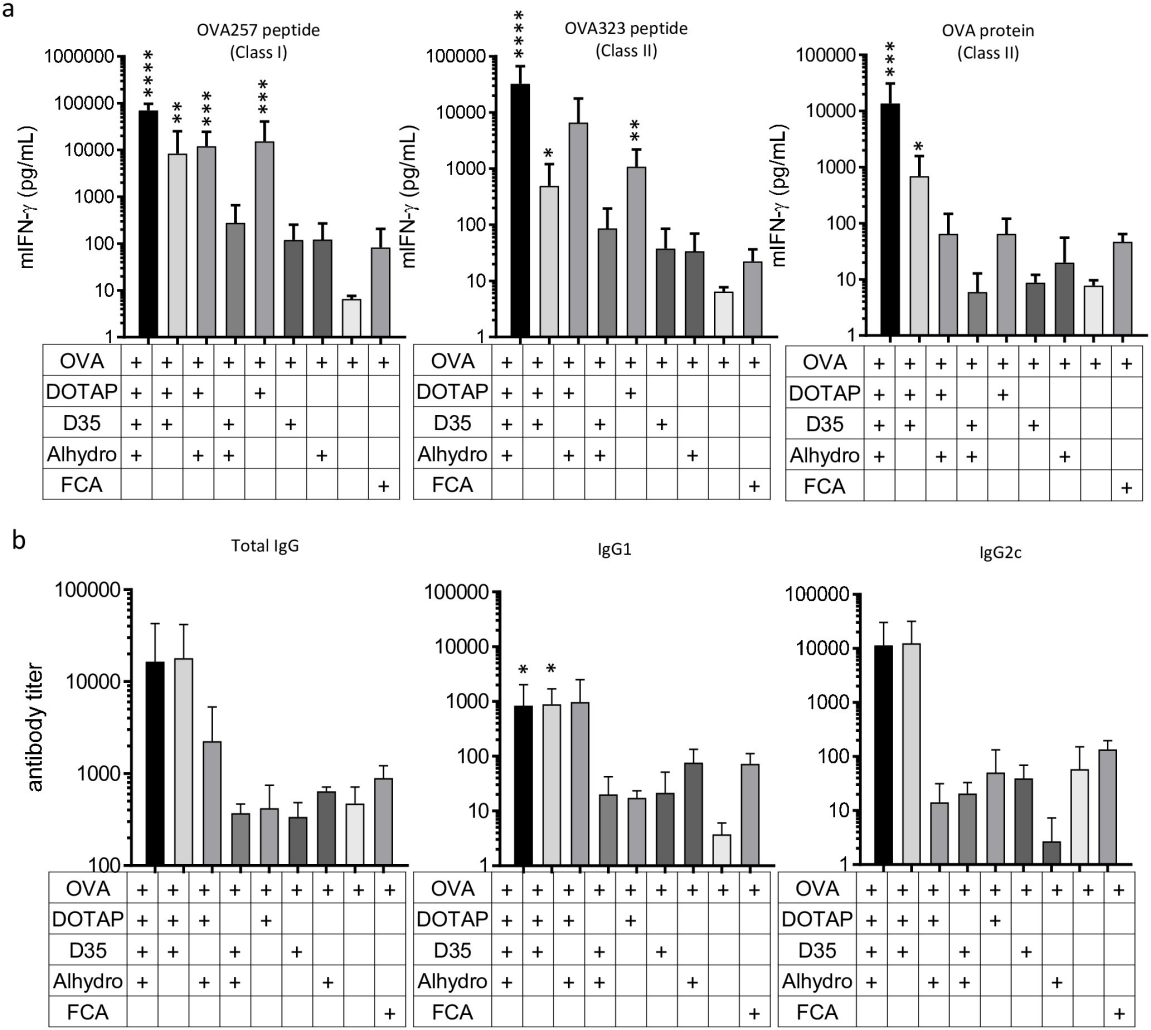
OVA/DOTAP/D35/Alhydrogel immunization induces strong T cell and antibody responses. C57BL/6J mice were immunized i.d. with OVA and the indicated adjuvant combinations at the tail base. OVA (10 μg), DOTAP (100 μg), D35 (10 μg), and Alhydrogel (40 μg) were mixed in a 1:1 mixture of 5% glucose and phosphate-buffered saline (PBS)(Glu/PBS buffer). After 7 days of immunization, splenocytes and serum were collected. The splenocytes were stimulated in vitro with OVA257-264 peptide that induces MHC class I restricted response (CD8^+^ T cell response), OVA323-339 peptide, or OVA protein that induces MHC class II restricted response (CD4^+^ T cell response). (a) After 24 h, the concentration of mouse IFN-γ in the culture supernatant was measured by ELISA. (b) The OVA-specific antibody titers of total IgG, IgG1, and IgG2c were determined by ELISA after 7 days of the single immunization. The bar graph indicates the mean + SD of cytokine concentration observed in six mice per group (a) and antibody titer calculated from three mice per group (b). Statistical significance compared with OVA only is shown by asterisks in the bar graph; *p < 0.05, **p < 0.01, ***p < 0.001, ****p < 0.0001.

Previously, we developed a DOTAP/D35 adjuvant to efficiently induce T cell responses against protein antigens, such as OVA, in mice [26]. We observed that both the microfluidic lipid particle preparation of DOTAP (DOTAP-Nano) and the DOTAP-antigen interaction are two important factors for DOTAP-based lipid particle adjuvants. The addition of Type A CpG D35 to DOTAP-Nano further enhanced the efficacy, especially for the induction of MHC class II restricted CD4^+^ T cell response [26]. To further improve DOTAP/D35 adjuvant efficacy, we examined the addition of Alhydrogel (aluminum hydroxide), an aluminum salt adjuvant that is popular and clinically used in many vaccines, in a DOTAP/D35 based adjuvant system. Induction of T cell and antibody responses were evaluated in mice. The combination of DOTAP/D35/Alhydrogel induced strong T cell responses 7 days following an immunization with 10 μg of OVA antigen (Fig 1a). Compared to OVA antigen only, OVA/DOTAP/D35/Alhydrogel significantly induced 1000-times more IFN-γ production by both CD8 and CD4 T cells from immunized splenocytes. It also induced 100-times more IFN-γ production than FCA. FCA is one of the strongest T cell responses inducing experimental adjuvants in animals (Fig 1a). Compared to immunization with OVA/DOTAP/D35, which induced strong T cell responses, the addition of Alhydrogel to OVA/DOTAP/D35 further increased T cell responses by approximately 10 times (Fig 1a). Furthermore, OVA/DOTAP/D35/Alhydrogel immunization induced IgG2c dominant OVA-specific antibody responses 7 days after immunization to a level comparable to that of OVA/DOTAP/D35 immunization (Fig 1b). OVA/DOTAP/D35/Alhydrogel immunization induced evident and rapidly increased average antibody titers were detected 7 days following immunization with OVA/DOTAP/D35/Alhydrogel and OVA/DOTAP/D35, although total IgG and IgG2c production was not significantly increased compared with OVA only immunization (Fig 1b). At this time, the antigen-specific antibody titer was usually barely detected using conventional adjuvant. These results suggest that the DOTAP/D35/Alhydrogel combination is an adjuvant that is highly efficient at inducing T cell and antibody immune responses.

### Type A CpG D35 is better than other types of CpG ODNs as a component of the combination adjuvant

**Fig 2:**
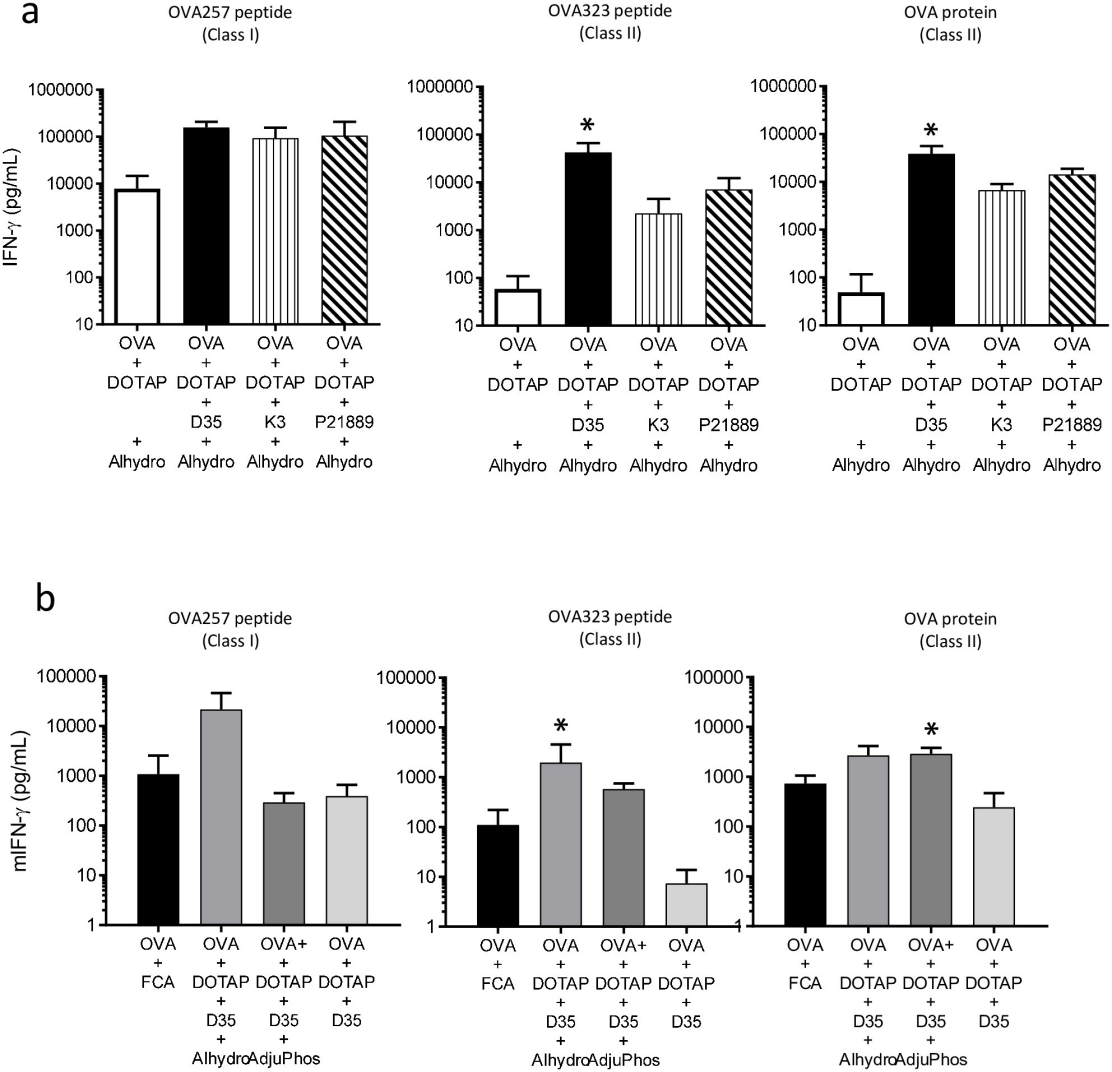
Type A CpG D35 and Alhydrogel are better components for the combination adjuvant to induce T cell responses. C57BL/6J mice were immunized i.d. with OVA and a combination adjuvant, as indicated at the tail base. OVA (10 μg), DOTAP (100 μg), CpG (D35, K3, or P21889; each 10 μg), and aluminum salt (Alhydrogel or AdjuPhos; each 40 μg) in Glu/PBS buffer were used. Seven days after immunization, splenocytes were collected and stimulated with OVA257-264 peptide to induce CD8^+^ T cell response, and OVA323-339, or OVA protein to induce CD4^+^ T cell response. After 24 h, the concentration of mouse IFN-γ in the culture supernatant was measured by ELISA. (a) Three different CpG comparisons were performed. (b) Comparison of Alhydrogel and Adjuphos in Glu/PBS buffer. The bar graph indicates the mean + SD of cytokine concentrations derived from three mice per group. Statistical significances compared with OVA/DOTAP/D35 (without Alhydrogel or Adjuphos) are shown by asterisks in the bar graph; *p < 0.05.

Next, we tested whether the inclusion of different types of CpG ODNs affected the combination adjuvant induced T cell responses. In this study, we used K3 as the B-type or P21889 as the P-type CpG ODNs. Mice were immunized with OVA/DOTAP/Alhydrogel using either D35, K3, or P21889. For MHC class I restricted OVA-specific CD8^+^ T cell responses, D35, K3, and P21889 comparably enhanced the responses by approximately 10 times compared to the absence of CpG ODN, but the difference was not reached significant (Fig 2a; left). For MHC class II restricted OVA-specific CD4^+^ T cell responses, OVA/DOTAP/D35/Alhydrogel showed significantly better CD4^+^ T cell response induction than OVA/DOTAP/Alhydrogel (i.e., without D35), and substantially (but not significantly) higher CD4^+^ T cell responses than K3-and P21889 containing adjuvants (Fig 2a; middle and right). This result suggested that the addition of any type of CpG enhanced the adjuvanticity. However, Type A CpG D35 showed overall better CD8^+^ and CD4^+^ T cell response induction compared to other types of CpG ODNs.

### Alhydrogel is better than AdjuPhos as a component of the combination adjuvant for OVA antigen

We also compared different types of aluminum salts as components of the combination adjuvant. The aluminum hydroxide Alhydrogel is usually used as an adjuvant for vaccination with negatively charged protein antigen, because the positive charge of the Alhydrogel in the neutral pH buffer allows electrostatic binding of negatively charged antigens to Alhydrogel, which is necessary for the adjuvant effect [10]. The aluminum phosphate AdjuPhos is used as an adjuvant for positively charged protein antigens with similar electrostatic interactions [10]. To test whether these electrostatic features affect immune induction, mice were immunized with OVA/DOTAP/D35 using either Alhydrogel or AdjuPhos. Substantially stronger (but not significant) OVA-specific CD8^+^ T cell responses were induced with OVA/DOTAP/D35/Alhydrogel compared to those induced by OVA/DOTAP/D35/AdjuPhos (Fig 2b; left). Alhydrogel and AdjuPhos induced significantly better CD4^+^ T cell responses than OVA/DOTAP/D35 immunization for OVA323 or OVA protein, respectively (Fig 2b; middle and right). However, the difference between Alhydrogel and AdjuPhos in CD4^+^ T cell responses was not obvious compared to that observed in CD8^+^ T cell responses (Fig 2b; left).

### Interaction between antigen and aluminum salt is an important factor for the adjuvanticity of the combination adjuvant

**Fig 3:**
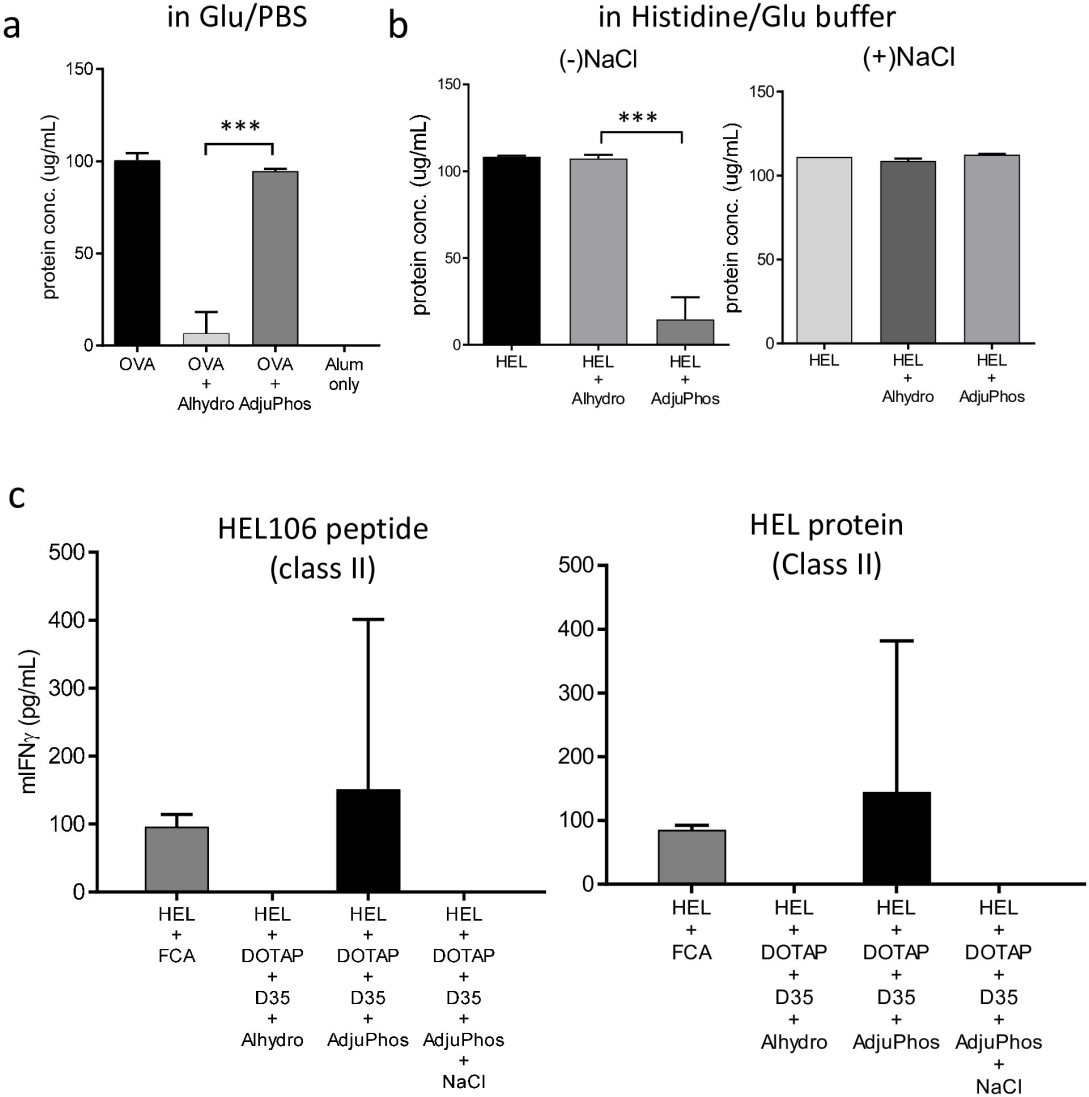
The interaction of the antigen and aluminum salt is important for the induction of T cell response with the combination adjuvant. OVA (10 μg), OVA plus Alhydrogel (40 μg), and OVA plus AdjuPhos (40 μg) in Glu/PBS were mixed and centrifuged at 5000 rpm for 3 min. The protein concentration in the supernatant was measured using a Qubit Protein Assay Kit. HEL (10 μg) only, HEL plus Alhydrogel (40 μg), HEL plus AdjuPhos (40 μg) in 20 mM histidine buffer (pH 6.5)(Glu/His buffer) (a) or in 20 mM histidine with 150 mM NaCl buffer (pH 6.5)(Glu/His+NaCl buffer) (b) were assayed in a similar manner. The bar graph indicates the mean ± SD of triplicate experiments. Statistical significance is indicated by an asterisk in the bar graph; ***p < 0.001(parametric unpaired t-test). (c) BALB/c mice were immunized with the indicated combination adjuvant. HEL (10 μg), DOTAP (100 μg), D35 (10 μg), and Alhydrogel or AdjuPhos (40 μg) in a 1:1 mixture of 5% glucose and histidine buffer (Glu/His) were used. As a control, HEL (10 μg) was also immunized with the FCA emulsion (see Methods). The AdjuPhos group also used as another control with 0.15 M NaCl addition. Seven days after immunization, splenocytes were collected and stimulated with HEL107-116 peptide or HEL protein to induce CD4^+^ T cell response. After 24 h, the concentration of mouse IFN-γ in the culture supernatant was measured by ELISA. The bar graph indicates the mean + SD of cytokine concentrations derived from three mice per group. The data are representative of at least two independent experiments with similar results.

T cell response induction, especially the difference in CD8^+^ T cell response induction between Alhydrogel and AdjuPhos, prompted us to examine the physical binding between OVA antigen and these aluminum salts. To test the protein absorption on the aluminum salt, antigen protein and each type of aluminum salt were mixed and centrifuged to separate the aluminum binding protein. In Glu/PBS, virtually no free OVA antigen was detected in the supernatant of the OVA and Alhydrogel mixture (Fig 3a). In contrast, almost all OVA antigens remained in the supernatant of the OVA and AdjuPhos mixture, suggesting that OVA did not bind to AdjuPhos in Glu/PBS (Fig 3a). This result was consistent with the fact that OVA antigen has a negative charge and Alhydrogel has a positive charge, while AdjuPhos has a negative charge in neutral pH buffer including Glu/PBS. Therefore, Alhydrogel and OVA physically interact with Glu/PBS via electrostatic forces.

We also tested another model antigen, HEL, which is positively charged in a neutral pH buffer. A similar experiment was performed in 20 mM histidine buffer, as NaCl in PBS prevents interaction between HEL and AdjuPhos interaction [29, 30]. As expected, HEL antigen was not absorbed on Alhydrogel, and HEL was completely absorbed on AdjuPhos in histidine buffer without NaCl (Glu/His) (Fig 3b; left). We also confirmed that HEL was not absorbed by AdjuPhos in the presence of 0.15 M NaCl (Glu/His with NaCl) (Fig 3b; right).

Based on these in vitro binding data, we immunized BALB/c mice with HEL plus combination adjuvants containing Alhydrogel, AdjuPhos, or AdjuPhos in the presence of 0.15 M NaCl. All samples were prepared in 20 mM histidine buffer (Glu/His buffer). We also included HEL plus FCA immunization as a positive control. HEL/DOTAP/D35/AdjuPhos immunization elicited detectable levels of CD4^+^ T cell responses against HEL106-117/I-E^d^ peptide or whole HEL protein, comparable to HEL/FCA immunization (Fig 3c). CD4^+^ T cell responses in both HEL/FCA and HEL/DOTAP/D35/AdjuPhos were detected. However, the responses were generally weak and highly variable among individuals, and were not statistically significant. Interestingly, HEL/DOTAP/D35/Alhydrogel or HEL/DOTAP/D35/AdjuPhos with NaCl immunization did not induce any detectable T cell responses (Fig 3c).

Notably, HEL did not contain CD8^+^ T cell epitope peptides in BALB/c(H-2d) mice. To examine CD8^+^ T cell response induction in response to HEL, we also immunized C57BL/6 (H-2b) mice, which have been reported to elicit CD8^+^ T cell responses against HEL23-31/H-2Db. However, we could not detect any IFN-γ secretion in response to HEL23-31/H-2Db and HEL protein after immunization with HEL (10 μg) plus DOTAP/D35/AdjuPhos in C57BL/6 mice. Therefore, we could not evaluate CD8^+^ T cell responses using the HEL antigen immunization model.

Taken together, these results suggest that for our developed combination adjuvant, the interaction between aluminum salt and vaccine antigen is important to induce T cell responses against vaccine antigens.

### Adjuvant component and antigen dose-dependent T cell response induction

We also examined the dose-effect of each vaccine component, including the OVA antigen, DOTAP, D35, and Alhydrogel. Two sequential experiments were conducted. These experiments required multiple group comparisons, and we could only use two mice per group. This prevented statistically significant calculation in these experiments. However, a trend was still evident for the immune responses. In experiment 1, the amounts of OVA (10 μg) and D35 (10 μg) were fixed, and different amounts of the combination of DOTAP (20, 40, 60, 80, and 100 μg) and Alhydrogel (20, 40, 60, 80 μg) were examined concerning MHC class I restricted CD8^+^ and MHC class II restricted CD4^+^ T cell response induction. Overall, increasing the dose of Alhydrogel increased both CD8^+^ and CD4^+^T cell responses (S1a Fig; left). Increasing the DOTAP dose also increased CD8^+^ T cell responses (S1a Fig; right). The inclusion of more than 60 μg of DOTAP was preferable (S1a Fig; right), especially for the induction of a strong CD8^+^ T cell response. In contrast, the induction of CD4^+^ T cell response was not affected by the DOTAP dose (S1a Fig; right).

In experiment 2, the amounts of Alhydrogel (80 μg) and DOTAP (80 μg) were fixed, and different amounts of the combination of OVA (0.1, 1, 10 μg) and D35 (1, 10, 100 μg) were examined concerning the induction of CD8^+^ and CD4^+^ T cell responses. CD8^+^ T cell response induction was strongly dependent on the amount of OVA (S1b Fig; left); 0.1μg of OVA immunization barely induced CD8^+^ T cell responses. In contrast, CD4^+^ T cell responses were much less dependent on the OVA dose (S1b Fig; left). Even 0.1μg of OVA induced detectable levels of CD4+ T cell responses when a sufficient amount of D35 was included in the formulation. CD8^+^ T cell responses were not affected by an increase in the amount of D35. However, strong CD4^+^ T cell responses were clearly detected with 10 μg and 100 μg of D35 (S1b Fig; right). The use of 1 μg of D35 induced only weak CD4^+^ T cell responses. These results suggest that increasing amounts of OVA, DOTAP, and Alhydrogel were linked with stronger CD8^+^ T cell response induction, while increased D35 resulted in a stronger CD4^+^ T cell response induction. Our initial formulation used in the experiment described in Fig 1, including OVA (10 μg), D35 (10 μg), DOTAP (100 μg), and Alhydrogel (40 μg), was an efficient formulation for this adjuvant system.

### Administration route affects vaccine efficacy

We also examined the different immunization routes with the formulation of OVA (10 μg), DOTAP (100 μg), D35 (10 μg), and Alhydrogel (40 μg) in Glu/PBS buffer. Tail base i.d. immunization induced consistent and better (but not statistically significantly) CD8^+^ and CD4^+^ T cell responses than other immunization routes, including intravenous, subcutaneous, intraperitoneal, and intramuscular (S2 Fig). The i.d. route at the tailbase used in most of our experiments was the most efficient for our adjuvant system.

### Importance of mutual physical interaction among antigen and other components

The interaction between antigen and DOTAP was important for the adjuvanticity of our previously developed DOTAP/D35 adjuvant [26]. This interaction was also affected by buffer conditions [26]. The results suggested that the interaction between the antigen and aluminum salt was also important for the enhanced adjuvanticity of DOTAP/D35/aluminum salt combination adjuvant (Figs 2b and 3). Therefore, we performed a detailed examination of the interaction between antigen and adjuvant components with four different buffer conditions, including Glu/PBS, Glu/His, Glu/His with NaCl, and MES as described in the Methods. Our previous study [26] and this study (Fig 3b) demonstrated that buffer conditions also substantially affect the interactions among vaccine components.

**Fig 4:**
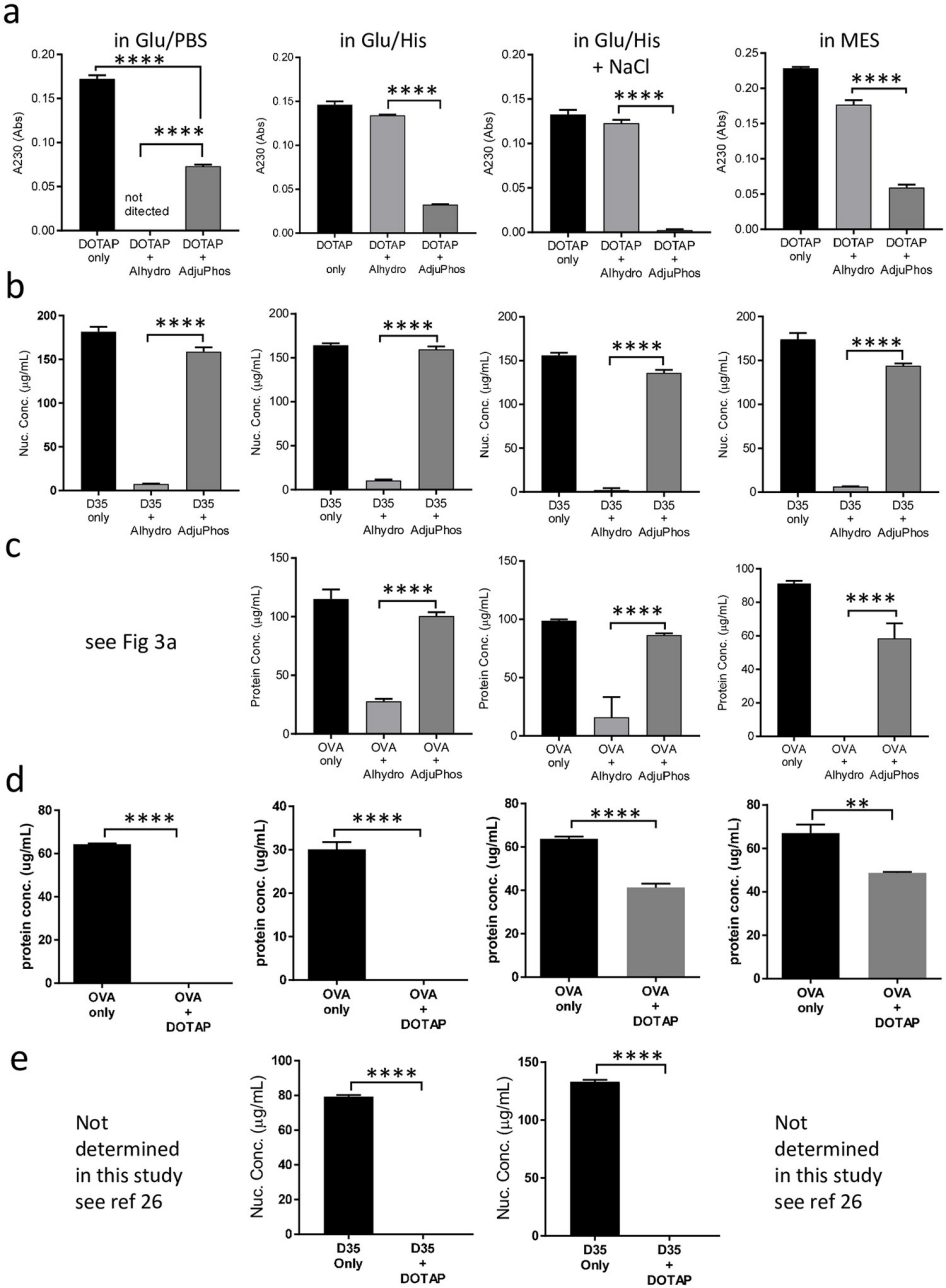
Interactions between adjuvant components in different buffers. The two-component interactions were examined using four different buffers. OVA (10 μg), DOTAP (100 μg), D35 (10 μg), and Alhydrogel (40 μg) were used. Two components were mixed in 100 μL of the total volume and examined as described in the Methods section. (a) DOTAP vs. Alhydrogel/AdjuPhos interaction (b) D35 vs. Alhycrogel/AdjuPhos interaction (c) OVA vs. Alhycrogel/AdjuPhos interaction (d) OVA vs. DOTAP interactions (e) D35 vs. DOTAP interaction. The bar graph indicates the mean ± SD of triplicates. Statistical significance is indicated by an asterisk in the bar graph; **p < 0.01, ***p < 0.001, ****p < 0.0001 (parametric unpaired t-test).

We first examined the interactions between DOTAP and aluminum salt using four different buffer conditions (Fig 4a). DOTAP and the indicated aluminum salts were mixed using these different buffer conditions. After centrifugation for the sedimentation of the aluminum salt and the aluminum binding DOTAP, the supernatant was examined for the remaining DOTAP content. During the development of this assay, we noticed that the A230 value of the absorbance meter was proportional to the amount of supernatant containing DOTAP (S3 Fig). In the Glu/PBS buffer, DOTAP was completely absorbed by the alhydrogel (Fig 4a). DOTAP was almost completely absorbed by AdjuPhos in Glu/His with NaCl (Fig 4a). Partial absorption was observed for DOTAP and AdjuPhos in Glu/PBS, Glu/His, and MES (Fig 4a). In contrast, very weak or almost no absorption was detected for DOTAP and Alhydrogel in Glu/His, Glu/His with NaCl, and MES (Fig 4a).

Second, we examined the interaction between D35 and aluminum salt. After mixing and centrifugation, the amount of D35 in the supernatant was measured using he absorbance at 280 nm. D35 was almost completely absorbed by the Alhydrogel, irrespective of the buffer conditions examined (Fig 4b). In contrast, D35 was not absorbed by AdjuPhos, irrespective of the buffer conditions examined (Fig 4b).

Third, the interaction between OVA and aluminum salt was similarly examined by the Qubit protein assay, as shown in Fig 3a and 3b. OVA was mostly absorbed by Alhydrogel in Glu/His and Glu/His with NaCl buffer, and completely absorbed in MES buffer (Fig 4c). Irrespective of the buffers, OVA was not absorbed by AdjuPhos (Figs 3a and 4c).

Fourth, the interactions between DOTAP and OVA were examined. DOTAP and OVA showed strong binding in the Glu/PBS and Glu/His buffers (Fig 4d). DOTAP and OVA partially interacted with Glu/His with NaCl and MES (Fig 4d).

Fifth, we reported that D35 strongly interacts with DOTAP in Glu/PBS and MES buffer conditions [26]. We tested other buffer conditions and observed that D35 was strongly bound to DOTAP in Glu/His and Glu/His with NaCl (Fig 4e).

The interactions between these components are schematically summarized in S4 Fig.

### Differences in interactions between various components affect T cell response induction

**Fig 5:**
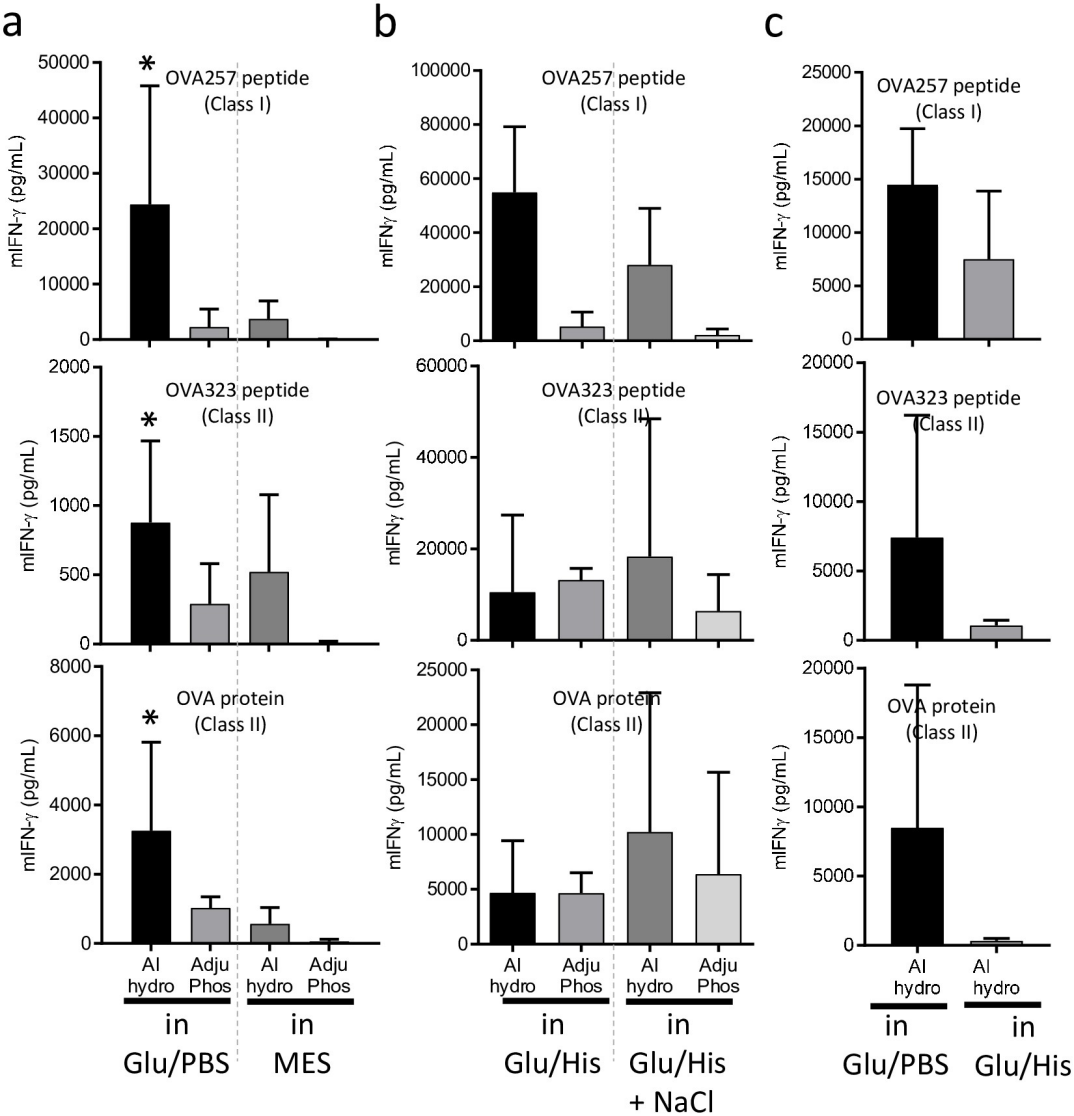
Induction of T cell response by different aluminum salt and buffers. C57BL/6J mice were immunized i.d. with OVA plus the indicated combination adjuvants and buffers at the tail base. OVA (10 μg), DOTAP (100 μg), D35 (10 μg), and Alhydrogel (40 μg) in the indicated buffers were used. Seven days after immunization, splenocytes were stimulated in vitro with OVA257-264 peptide that induced MHC class I restricted response (CD8^+^ T cell response), and OVA323-339 peptide, or OVA protein that induced MHC class II restricted response (CD4^+^ T cell response). After 24 h, the concentration of mouse IFN-γ in the culture supernatant was measured by ELISA. (a) Comparison of Glu/PBS and MES buffers (b) Comparison of Glu/His and Glu/His + NaCl solutions. (c) Comparison of Glu/PBS and Glu/His buffers. The bar graph indicates the mean + SD of cytokine concentrations derived from three mice per group. Statistical significances compared with Alhydrogel in Glu/PBS and AdjuPhos in MES (in panel (a)) were detected and are shown by asterisks in the bar graph; *p < 0.05. Other comparisons did not reach statistical significance (non-parametric test).

We further examined whether the differences in the interactions between these components could affect T cell response induction in vivo. We conducted two sets of experiments. The first experiment compared two buffer conditions, Glu/PBS or MES, for the Alhydrogel and AdjuPhos aluminum salts. OVA (10 μg), D35 (10 μg), and DOTAP (100μg) were mixed with either Alhydrogel (40μg) or AdjuPhos (40μg) in the Glu/PBS or MES buffer. After 7 days of immunization, OVA-specific T cell responses of splenocytes were evaluated. Alhydrogel in Glu/PBS induced the strongest T cell responses (Fig 5a). AdjuPhos in MES showed almost no adjuvant effect and, interestingly, was associated with one of the weakest interactions of each component (S4 Fig). In the second experiment, a similar comparison was performed with the Glu/His or Glu/His+NaCl buffer. Alhydrogel in Glu/His showed the strongest T cell responses, although they were not statistically significant (Fig 5b). Especially for Class I responses, AdjuPhos did not induce potent T cell responses in either buffer. The experimental results varied, especially for T cell responses, since TCR recombination is individual, and the resultant T cell repertoires are quite different, even in twins [31–33]. Hence, it was difficult to compare the results of two independent experiments using absolute IFN-γ measurement values. Therefore, we performed a third experiment to directly compare Alhydrogel in Glu/PBS and Alhydrogel in Glu/His. The T cell response was induced by Alhydrogel in Glu/PBS compared to that in Glu/His (Fig 5c). Although not significant, Alhydrogel in Glu/PBS showed better overall T cell responses than Alhydrogel in Glu/His (Fig 5c), which was associated with the lack of interaction between DOTAP and Alhydrogel in Glu/His (Fig 4a and S4 Fig). The overall T cell response induction appeared to be associated with an interaction of each component. Alhydrogel in Glu/PBS, which showed the strongest mutual interaction among components (S4 Fig), demonstrated the strongest induction of T cell response (Fig 5a and 5c). In contrast, AdjuPhos in MES, which showed the weakest mutual interaction among components (S4 Fig), demonstrated almost no T cell response induction (Fig 5a).

### Differences in antigen distribution pattern between adjuvant formulations in the presence and absence of DOTAP

**Fig 6:**
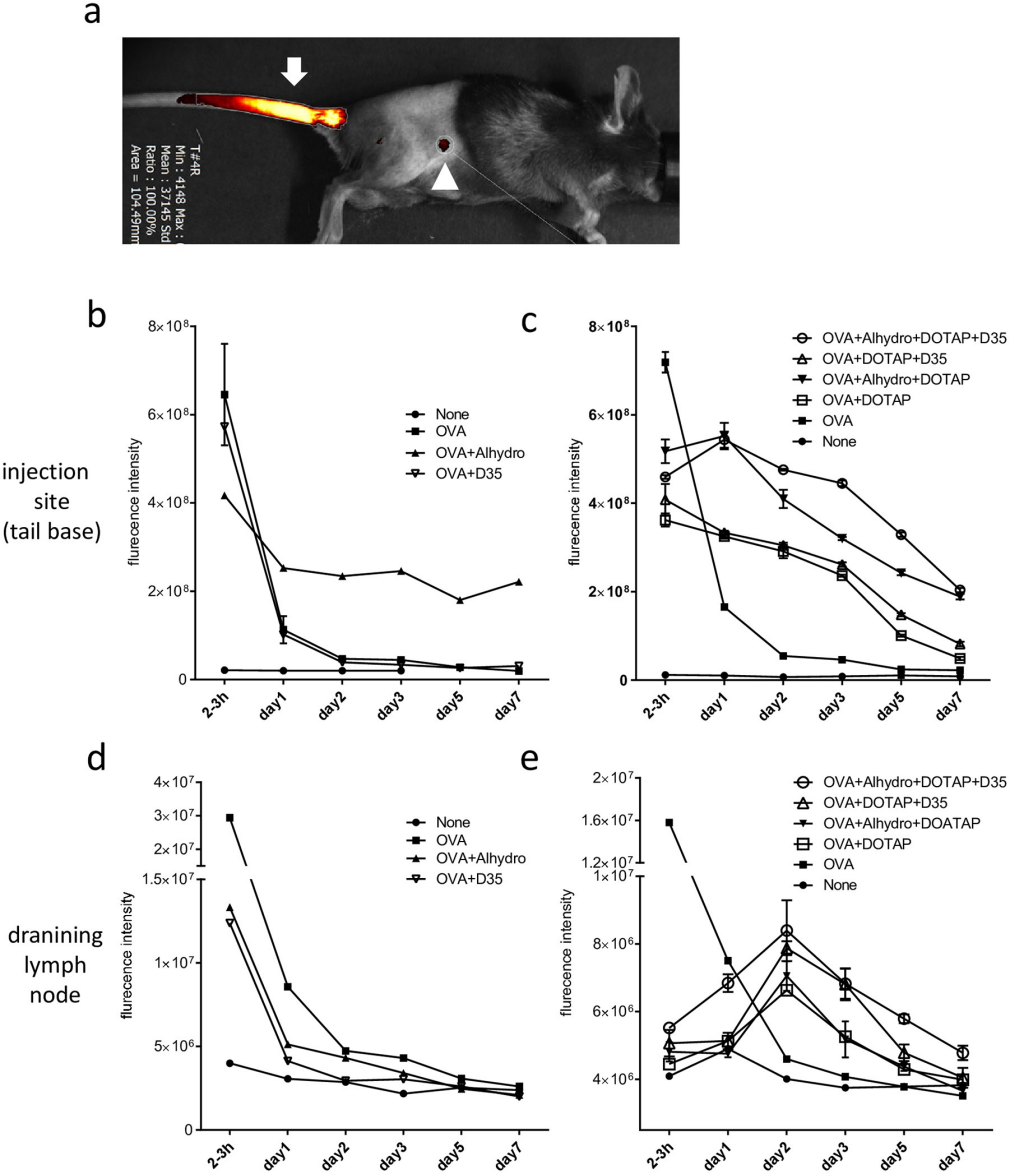
Biodistribution of OVA antigen in vivo after injection of different formulation of adjuvants. C57BL/6J mice were administered Alexa647-labeled OVA (10 μg) or the indicated adjuvant formulations via the tail base. For combination, Alexa647-OVA (10 μg), DOTAP (100 μg), D35 (10 μg), and Alhydrogel (40 μg) in the Glu/PBS buffer were used. The fluorescence intensity of Alexa647 was measured every 1–2 days. (a) Representative image of Alexa647-labeled OVA detection using the VISQUE^®^ InVivo imager. The arrow indicates the signal at the injection site, and the arrowhead indicates the signal observed in the draining lymph node. DOTAP containing formulations were examined; (b)(d) None(Glu/PBS buffer only), OVA, OVA+Alhydrogel, OVA+D35. DOTAP containing formulations were examined; (c)(e) OVA+Alhydrogel+DOTAP+D35, OVA+DOTAP+D35, OVA+alhydrogel+DOTAP, OVA+DOTAP, OVA, None. The fluorescent intensities at the injection site (b)(c) and inguinal lymph node (d)(e) were measured using the VISQUE^®^ InVivo imager.

We examined the antigen distribution kinetics in vivo. Antigen distribution after immunization was monitored using AlexaFluor 647 labeled OVA antigen and VISQUE^®^ InVivo imager (Fig 6a; a representative image of VISQUE^®^ InVivo imager; the arrow indicates the signal at the injection site (tail base; i.d.), and the arrowhead indicates the signal observed in the draining lymph node (inguinal lymph node)). Mice were immunized i.d. at the tail base with AlexaFluor 647 labeled OVA antigen (10 µg) with various DOTAP/D35/Alhydrogel combination adjuvants in Glu/PBS buffer. The fluorescence signals were observed over time. Two sets of experiments were conducted. In the first experiment, we examined OVA, OVA + Alhydrogel, and OVA+D35 (Fig 6b and 6d). Very strong signals were detected at the injection site 2–3 h after injection, and then quickly disappeared within 2 days (Fig 6b). The OVA in OVA+Alhydrogel was retained at the injection site up to day 7, indicating the depot effect of Alhydrogel on OVA. Similarly, at the draining lymph node, OVA signals peaked 2–3 h after injection and then decreased almost to the background level after 2 days (Fig 6d).

In the second experiment, we examined other adjuvant combinations containing DOTAP as a formulation component. Compared to the results of experiment 1, the OVA antigen of DOTAP containing formulations was relatively well retained at the injection site. The corresponding signals gradually decreased up to day 7 (Fig 6c). Interestingly, at the draining lymph node, the OVA antigen in the DOTAP containing formulation temporarily peaked on day 2 after injection (Fig 6e). This type of antigen distribution has been shown to associate with antigen-containing dendritic cell migration from the injection site to the draining lymph node [34, 35], suggesting that the formulation containing DOTAP induced DC antigen uptake and migration.

## Discussion

The results demonstrated that the combination adjuvant consisting of DOTAP, D35, and aluminum salt acted as a potent T cell and antibody response inducing adjuvant for protein vaccine antigens. We examined the features of this combination adjuvant system using two commonly used model antigens, OVA and HEL. We observed that the electrostatic interaction between the antigen protein and aluminum salt is an important factor affecting adjuvanticity. For OVA antigen, the alhydrogel containing the combination adjuvant showed better adjuvanticity than AdjuPhos (Fig 2b). For the HEL antigen, AdjuPhos was better than Alhydrogel (Fig 3c). This rule has been well recognized for antibody response induction by an aluminum salt adjuvant [36]. Our data demonstrated that antigen and aluminum salt interactions are also important factors for T cell response induction [37]. Hansen et al. also reported that the interaction strength between antigen and aluminum salt influenced the induction of T cell response, and a very strong interaction reduced T cell responses. This antigen adsorption strength issue in our system needs to be examined in future experiments. Nevertheless, the requirement of antigen and aluminum salt interaction also suggests that aluminum salt is an important component of our combination adjuvant system.

Our data showed that OVA/DOTAP/D35/Alhydrogel in Glu/PBS buffer produced the strongest induction of T cell responses (Fig 5c) associated with the strongest mutual interactions between antigens and each component (S4 Fig). This finding indicates that complex formation is critical for our DOTAP/D35/aluminum complex adjuvant. In contrast, many adjuvants, including Advax, MF59, AS01, AS03, and ISCOM do not necessarily require antigen and adjuvant binding for their adjuvanticity [38–43]. These adjuvants induce local inflammation, which stimulates immune cell recruitment to the draining lymph node, where co-administrated antigens are taken up by dendritic cells (DCs) [44].

Induction of Alhydrogel and OVA antigen dose-dependent T cell responses was demonstrated in experiments performed with increasing doses of each component (S1a and S1b Fig left panel). The results also demonstrated the importance of the interaction between antigen and aluminum salt for adjuvanticity. The doses of the other two components, DOTAP and D35, produced distinct effects on CD8^+^ T cells and CD4^+^ T cell responses, respectively. Relatively large doses of DOTAP increased CD8^+^ T cell response induction, whereas CD4^+^ T cell response induction was not dependent on the DOTAP dose (S1a Fig; right panel). In contrast, D35 did not significantly influence CD8^+^ T cell response induction. However, an increased dose of D35 resulted in stronger CD4^+^ T cell responses (S1a Fig; right panel). These results suggest that DOTAP and D35 may act on different antigen presentation processes, leading to efficient CD4^+^ and CD8^+^ T cell response induction. Currently, the mechanism underlying the adjuvant activity of DOTAP is not fully understood [18, 19, 45]. However, the results of our biodistribution experiments suggested that DOTAP induced DC migration from the local injection site into the draining lymph node based on the delayed antigen increase in the draining lymph node with DOTAP containing adjuvant combinations on day 2 following immunization (Fig 6e). These results are consistent with the fact that the i.d. tail base route is the best route of immunization (S2 Fig). On the other hand, the signal of OVA only or OVA with Alhydrogel was highest 2–3 h after injection and rapidly (in the case of free OVA) or gradually (in the case of OVA with Alhydrogel) decreased at the injection site (Fig 6b). These findings suggest that without DOTAP, the injected OVA only dispersed the surrounding tissue and flowed away rapidly to the draining lymph node, in contrast to the above-mentioned DOTAP containing case. Of note, our in vivo imager analysis was not definitively quantitative. This was because of the complex molecular interaction between Alexa647 labeled OVA and other adjuvant components possibly influenced the excitation and emission efficiencies of the Alexa647 fluorescent dye. In addition, the in vivo imaging system is primarily a semi-quantitative method. Therefore, the interpretation of data in Fig 6 is limited to the differences in the peak time kinetics between adjuvants that containing DOTAP or do not.

DC migration has been shown to increase the immune response, especially the induction of the T cell response [34, 35]. To the best of our knowledge, this is the first study to report DOTAP induced antigen-containing DC migration. We believe that DC migration induction is an important mechanism of DOTAP-mediated adjuvant activity.

D35 is a type A CpG ODN and an agonist ligand of TLR9. In many adjuvants incorporating CpG ODNs, the addition of CpG to the adjuvant system usually enhances CD8^+^ T cell response more than the CD4^+^ T cell response [46, 47]. This tendency of immune-stimulatory nucleic acid incorporation-enhanced CD8^+^ T cell response is also applicable to a variety of TLR ligand incorporating CAF family adjuvants. Among the many different CAF family adjuvants, CAF05 and CAF09 include Poly IC, which efficiently enhances CD8^+^ T cell responses. However, CAF01 and CAF04 (base liposomal adjuvant without Poly IC to prepare CAF05 and CAF09, respectively) only induce CD4^+^ T cell responses [48–50]. CpG ODN incorporation into another adjuvant ISCOM was reported to similarly enhance CD8^+^ T cell responses [51]. In our combination adjuvant, incorporation of D35 did not significantly enhance CD8^+^ T cell responses compared to the absence of D35 (Fig 1a; OVA/DOTAP/D35 vs OVA/DOTAP). These results are consistent with those obtained in our previous study [26]. Incorporation of DOTAP itself already saturated CD8^+^ T cell responses, or D35 may have preferentially enhanced CD4^+^ T cell responses over CD8^+^ T cell responses in the combination of DOTAP/D35/Alhydrogel. These observations require further examination. Distinct DCs, such as DCIR2^+^ and CD8α^+^, preferentially present antigens to CD4^+^ and CD8^+^ T cells, respectively [52]. These DCs are positioned differently in the lymphoid organs [53–55], suggesting that each component dose may differentially affect the cellular biodistribution of antigens in vivo. This is an important hypothesis that requires examination in future experiments.

Our combination adjuvant DOTAP/D35/Alhydrogel is an efficient T cell response inducing adjuvant for negatively charged protein antigens, such as OVA. Changing the aluminum salt component to AdjuPhos led to the formation of DOTAP/D35/AdjuPhos, which is an effective adjuvant for positively charged protein antigens, such as HEL. This result demonstrated the suitability of our combination of DOTAP/D35/aluminum salt to a variety of protein antigens. This combination adjuvant induced strong immune responses, including T cell and antibody responses, within 7 days following single immunization, suggesting rapid and efficient induction of immunity.

Recently, we also examined and reported a DOTAP/D35/Alhydrogel combination adjuvant for a more clinically relevant protein antigen, HHV-6B tetrameric protein antigen [56]. HHV-6B causes exanthem subitum in early childhood, and some infants infected with HHV-6B virus develop encephalitis with poor prognosis. HHV-6B tetrameric protein antigen is currently in preclinical developmental phase evaluation as a preventive vaccine against HHV-6B infection. For this HHV-6B antigen, our DOTAP/D35/Alhydrogel combination adjuvant also induced efficient neutralizing antibody and Th1/Th2 balanced T cell responses for HHV-6B antigen in mice [56].

Hence, we believe that our combination adjuvant is a promising vaccine adjuvant candidate for other infectious diseases and cancers that require strong and rapid T cell response induction, although more comprehensive cytokine examination and mechanism research are required in the future.

## Supporting information

S1 Fig Antigen and adjuvant components dose-dependently induced T cell responses with the combination adjuvant. Experiment 1: (a, left x-axis) Alhydrogel (20–80 μg) or (a, right x-axis) DOTAP (20–100 μg) in Glu/PBS buffer was altered in combinations (n = 2, total 20 groups). The induction of a T cell response was examined by ELISA measuring IFN-gamma production. Experiment 2: (b, left x-axis) OVA (0.1–10 μg) or (b, right x-axis) D35 amount (1–100 μg) in Glu/PBS buffer were changed in combinations (n = 2, total nine groups). The T cell response induction was examined by ELISA. The same results are used in the left and right panels, and are rearranged as indicated. The bar graph indicates the mean + SD of cytokines derived from two mice per group.
(DOCX)

S2 Fig T cell immune response induced via different immunization routes. Mice were immunized with OVA (10 μg), DOTAP (100 μg), D35 (10 μg), and Alhydrogel (40 μg) in Glu/PBS buffer via the indicated routes, including the tail base (i.d.), intravenous (i.v.), subcutaneous (s.c.), intraperitoneal (i.p.), and intramuscular (i.m.) routes. After 7 days of immunization, splenocytes were stimulated with OVA-specific MHC class I (a) or class II (b) peptide or OVA whole protein (c) in vitro for 24 h. The secreted IFN-gamma levels were measured using ELISA. Each dot indicates the cytokine concentration derived from one mouse (three mice per group).
(DOCX)

S3 Fig The amount of DOTAP in the buffer solution measured using absorbance at 230 nm (A230). A230 of DOTAP (blue line) in 5% glucose solution or A230 of the supernatant after centrifugation of DOTAP + alum (red line) mixture. DOTAP in 5% glucose solutions showed high signals and low background signals at A230, as measured by the NanoDrop 2000 device. A similar profile was obtained using other buffers, including PBS, histidine buffer, and MES buffer.
(DOCX)

S4 Fig Schematic diagrams of vaccine component interactions in different buffers based on the data shown in Fig 4. The bold black line indicates a strong interaction. The gray dotted line indicates the intermediate interactions. No lines between the components indicated very weak or no interactions.
(DOCX)
