# Supplementary material for: Development of combination adjuvant for efficient T cell and antibody response induction against protein antigen
Source: PLoS One. 2021 Aug 2;16(8):e0254628. doi: 10.1371/journal.pone.0254628 (PMC8328330; doi:10.1371/journal.pone.0254628)
Supplement: S1 Fig — Experiment 1: (a, left x-axis) Alhydrogel (20–80 μg) or (a, right x-axis) DOTAP (20–100 μg) in Glu/PBS buffer was altered in combinations (n = 2, total 20 groups). The induction of a T cell response was examined by ELISA measuring IFN-gamma production. Experiment 2: (b, left x-axis) OVA (0.1–10 μg) or (b, right x-axis) D35 amount (1–100 μg) in Glu/PBS buffer were changed in combinations (n = 2, total nine groups). The T cell response induction was examined by ELISA. The same results are used in the left and right panels, and are rearranged as indicated. The bar graph indicates the mean + SD of cytokines derived from two mice per group. (DOCX) [file pone.0254628.s001.docx]

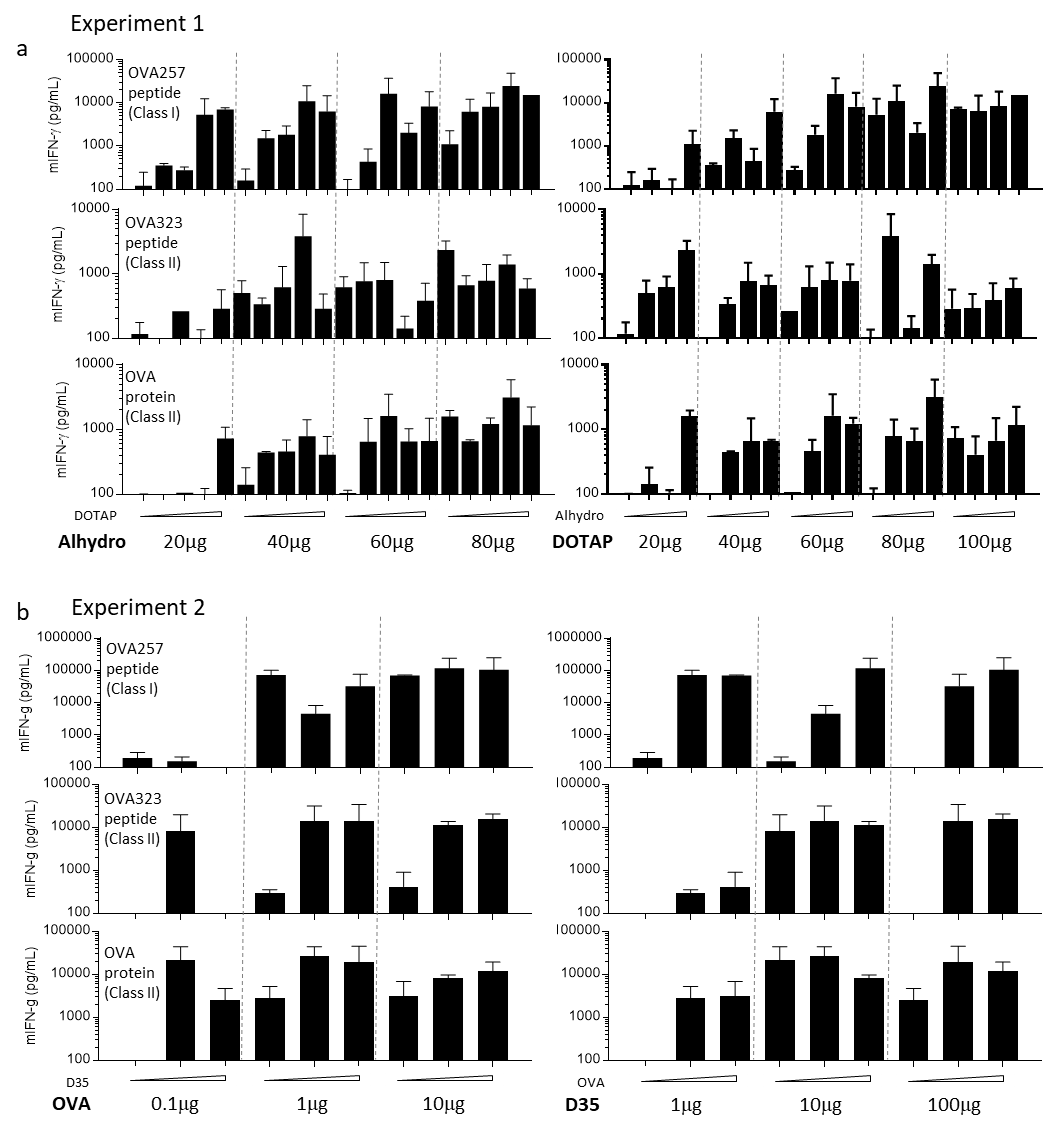


**S1 Fig. Antigen and adjuvant component dose-dependently induced T cell response with the combination adjuvant. Experiment 1:** (a, left x axis) Alhydrogel (20 – 80μg) or (a, right x-axis) DOTAP (20 – 100μg) in Glu/PBS buffer was altered in combinations (n=2, total 20 groups). The induction of a T cell response was examined by ELISA measuring IFN-gamma production. **Experiment 2:** (b, left x-axis) OVA (0.1 – 10μg) or (b, right x-axis) D35 amount (1–100μg) in Glu/PBS buffer were changed in combinations (n=2, total nine groups). The T cell response induction was examined by ELISA. The same results are used in the left panel and right panel, and are rearranged as indicated. The bar graph indicates the mean+SD of cytokines derived from two mice per group.
