# Supplementary material for: Development of combination adjuvant for efficient T cell and antibody response induction against protein antigen
Source: PLoS One. 2021 Aug 2;16(8):e0254628. doi: 10.1371/journal.pone.0254628 (PMC8328330; doi:10.1371/journal.pone.0254628)
Supplement: S2 Fig — Mice were immunized with OVA (10 μg), DOTAP (100 μg), D35 (10 μg), and Alhydrogel (40 μg) in Glu/PBS buffer via the indicated routes, including the tail base (i.d.), intravenous (i.v.), subcutaneous (s.c.), intraperitoneal (i.p.), and intramuscular (i.m.) routes. After 7 days of immunization, splenocytes were stimulated with OVA-specific MHC class I (a) or class II (b) peptide or OVA whole protein (c) in vitro for 24 h. The secreted IFN-gamma levels were measured using ELISA. Each dot indicates the cytokine concentration derived from one mouse (three mice per group). (DOCX) [file pone.0254628.s002.docx]

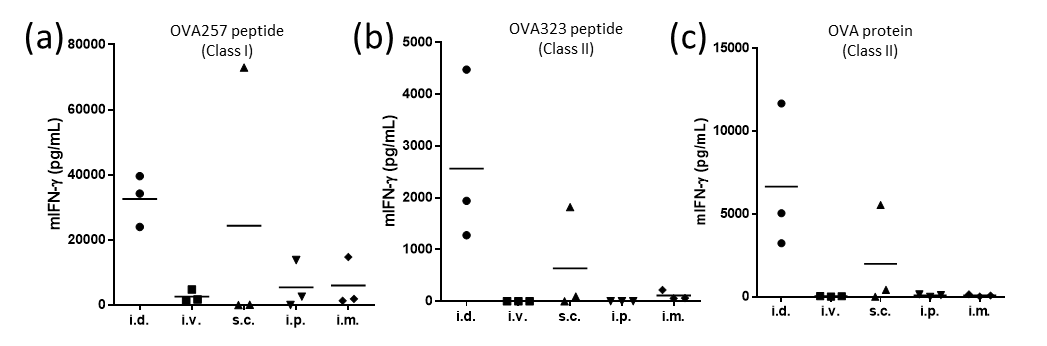


**S2 Fig.** **T cell immune response induced via different immunization routes** Mice were immunized with OVA(10μg)/DOTAP(100μg)/D35(10μg)/Alhydrogel(40μg) in Glu/PBS buffer via the indicated routes including tail base (i.d.), intravenous (i.v.), subcutaneous (s.c.), intraperitoneal (i.p.), and intramuscular (i.m.) routes. After 7 days of immunization, the splenocytes were stimulated with OVA-specific MHC class I (a) or class II (b) peptide or OVA whole protein (c) in vitro for 24 h. The secreted IFN-gamma was measured by ELISA. The each dot indicates the cytokine concentration derived from one mouse (three mice per group).
