# Supplementary material for: Development of combination adjuvant for efficient T cell and antibody response induction against protein antigen
Source: PLoS One. 2021 Aug 2;16(8):e0254628. doi: 10.1371/journal.pone.0254628 (PMC8328330; doi:10.1371/journal.pone.0254628)
Supplement: S3 Fig — A230 of DOTAP (blue line) in 5% glucose solution or A230 of the supernatant after centrifugation of DOTAP + alum (red line) mixture. DOTAP in 5% glucose solutions showed high signals and low background signals at A230, as measured by the NanoDrop 2000 device. A similar profile was obtained using other buffers, including PBS, histidine buffer, and MES buffer. (DOCX) [file pone.0254628.s003.docx]

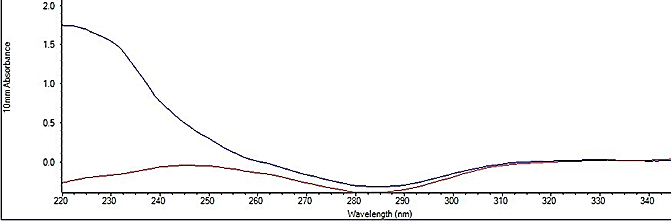


**S3 Fig. The amount of DOTAP in the buffer solution measured using absorbance at 230 nm (A230).** A230 of DOTAP (blue line) in 5% glucose solution or A230 of the supernatant after centrifugation of DOTAP + alum (red line) mixture. DOTAP in 5% glucose solutions showed high signals and low background signals at A230, as measured by the NanoDrop 2000 device. A similar profile was obtained using other buffers, including PBS, histidine buffer, and MES buffer.
