# Supplementary material for: Development of combination adjuvant for efficient T cell and antibody response induction against protein antigen
Source: PLoS One. 2021 Aug 2;16(8):e0254628. doi: 10.1371/journal.pone.0254628 (PMC8328330; doi:10.1371/journal.pone.0254628)
Supplement: S4 Fig — The bold black line indicates a strong interaction. The gray dotted line indicates the intermediate interactions. No lines between the components indicated very weak or no interactions. (DOCX) [file pone.0254628.s004.docx]

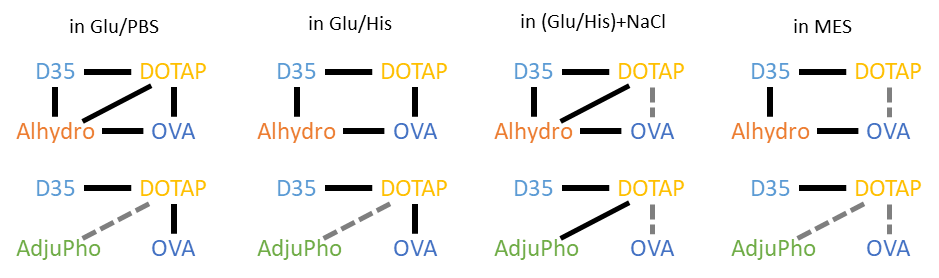


**S4 Fig. Schematic diagrams of vaccine component interactions in different buffers based on the data shown in Fig 4.** The black bold line indicates strong interaction. The gray dotted line indicates intermediate interaction. No lines between components indicate very weak or no interactions.
